# Addition of McKenzie Exercises to a Multimodal Physical Therapy Approach for Cervical Radiculopathy: A Case Report

**DOI:** 10.7759/cureus.32992

**Published:** 2022-12-27

**Authors:** Anthony Baumann, Laura Fisher

**Affiliations:** 1 Department of Rehabilitation Services, University Hospitals Cleveland Medical Center, Cleveland, USA; 2 Department of Physical Therapy, University of Michigan, Ann Arbor, USA

**Keywords:** mckenzie, physical therapy, exercise, neck pain, cervical radiculopathy

## Abstract

Cervical radiculopathy is a common subset of neck pain involving cervical nerve root irritation potentially resulting in numbness, radicular pain, and/or upper extremity weakness. The Neck Pain Clinical Practice Guidelines published by the American Physical Therapy Association does not support McKenzie exercises in isolation when treating cervical radiculopathy, but endorses a multimodal physical therapy approach for the management of cervical radiculopathy. The purpose of this case report is to exemplify the treatment of a patient with cervical radiculopathy utilizing McKenzie centralization exercises within a multimodal physical therapy approach for improved patient outcomes. The patient was a 49-year-old female with a past medical history of type 1 diabetes mellitus with a history of subacute cervical pain with left upper extremity radicular symptoms for four months consistent with cervical radiculopathy. Interventions included a multimodal physical therapy approach consisting of McKenzie cervical retraction exercises, thoracic manipulation, rib mobilizations, manual cervical traction, peripheral nerve mobilization, and scapular retraction with postural exercises. The patient received four visits over a five-week period with an emphasis on patient education and independence. Although McKenzie centralization exercises are not supported by the Neck Pain Clinical Practice Guideline (CPG) in isolation, adding these exercises to the supported recommendation of a multimodal physical therapy approach for cervical radiculopathy has promise.

## Introduction

Neck pain is a common musculoskeletal condition with up to 70% of the population experiencing neck-related symptoms throughout life [1,2]. The prevalence of neck pain among the general population increases with age and is most common in middle-aged women [1,2]. Cervical radiculopathy is a common subset of neck pain which is believed to result from various mechanisms causing cervical nerve root irritation and a wide variety of symptoms distal to the irritation site [3-7]. Common causes of cervical radiculopathy include cervical stenosis, spinal degenerative changes, and cervical disc herniation, which can cause nerve root compression and/or irritation [3,6,8]. Clinical symptoms of cervical radiculopathy often include neck pain with radiating pain into the upper extremity as well as dermatomal weakness, numbness, and/or strength deficits [1,6]. Cervical radiculopathy is believed to affect about 85 out of every 100,000 Americans each year and represents a considerable medical and economic burden [4]. Overall, the prognosis of cervical radiculopathy is favorable with up to 90% of patients responding positively to conservative treatment alone [4,9]. Along with pain medication, physical therapy (PT) is a commonly used conservative treatment for patients with cervical radiculopathy [3,4,6].

The McKenzie Method of Diagnosis and Treatment (MMDT) is an approach of PT that can be used for the treatment of spinal pain, especially with associated radicular symptoms [4,10]. A vital component of MMDT is the ability to centralize or decrease the patient’s referred or radicular symptoms through a series of repeated movements [4]. Although the concept of repeated motions and centralization have been shown to be effective in treating radicular symptoms associated with the lumbar spine, evidence is inconclusive for the use of specific, repeated motions for treating cervical radiculopathy [1].

According to the 2008 Neck Pain Clinical Practice Guideline (CPG) of the American Physical Therapy Association, “specific repeated movements or procedures to promote centralization are not beneficial in reducing disability when compared to other forms of interventions” [1]. In the 2017 revision of the Neck Pain CPG, the recommendation for treating neck pain with radiating pain is a multimodal approach utilizing “mechanical intermittent cervical traction, combined with other interventions such as stretching and strengthening exercises plus cervical and thoracic mobilization/manipulation” [1]. The current literature is conflicted on the benefits of MMDT for cervical pain. One study demonstrated that McKenzie exercises involving repeated movements did not demonstrate significantly superior outcomes compared to general exercise and a control group at long-term follow-up (12 months), but did show more rapid improvement in pain symptoms in the short-term (three weeks) [11]. Significantly, similar short-term results without significant long-term outcomes are seen among other studies that involve multimodal treatments and are not specific to studies regarding McKenzie exercises [11].

Some of the criticism regarding studies concerning conservative treatment for cervical radiculopathy is that all patients involved in the studies were treated in the same way without regard to specific unique patient presentations that could alter the plan of care [8]. The need for clinical reasoning continues to be evident as the 2017 revision of the Neck Pain CPG also recommends that “clinicians should monitor symptom irritability, and adjust treatment accordingly, when applying manual therapy and exercise approaches applied to patients with radicular pain” indicating the potential for using certain techniques when deemed appropriate mostly by patient presentation [1]. Overall, research addressing conservative management of cervical radiculopathy is lacking, little is known about the most effective treatments for cervical radiculopathy, and there has been little advancement on conservative treatments for cervical radiculopathy [1,8,9]. This case report exemplifies the treatment of a patient with cervical radiculopathy utilizing McKenzie centralization exercises within a multimodal physical therapy approach to improve patient outcomes.

## Case presentation

**Figure 1:**
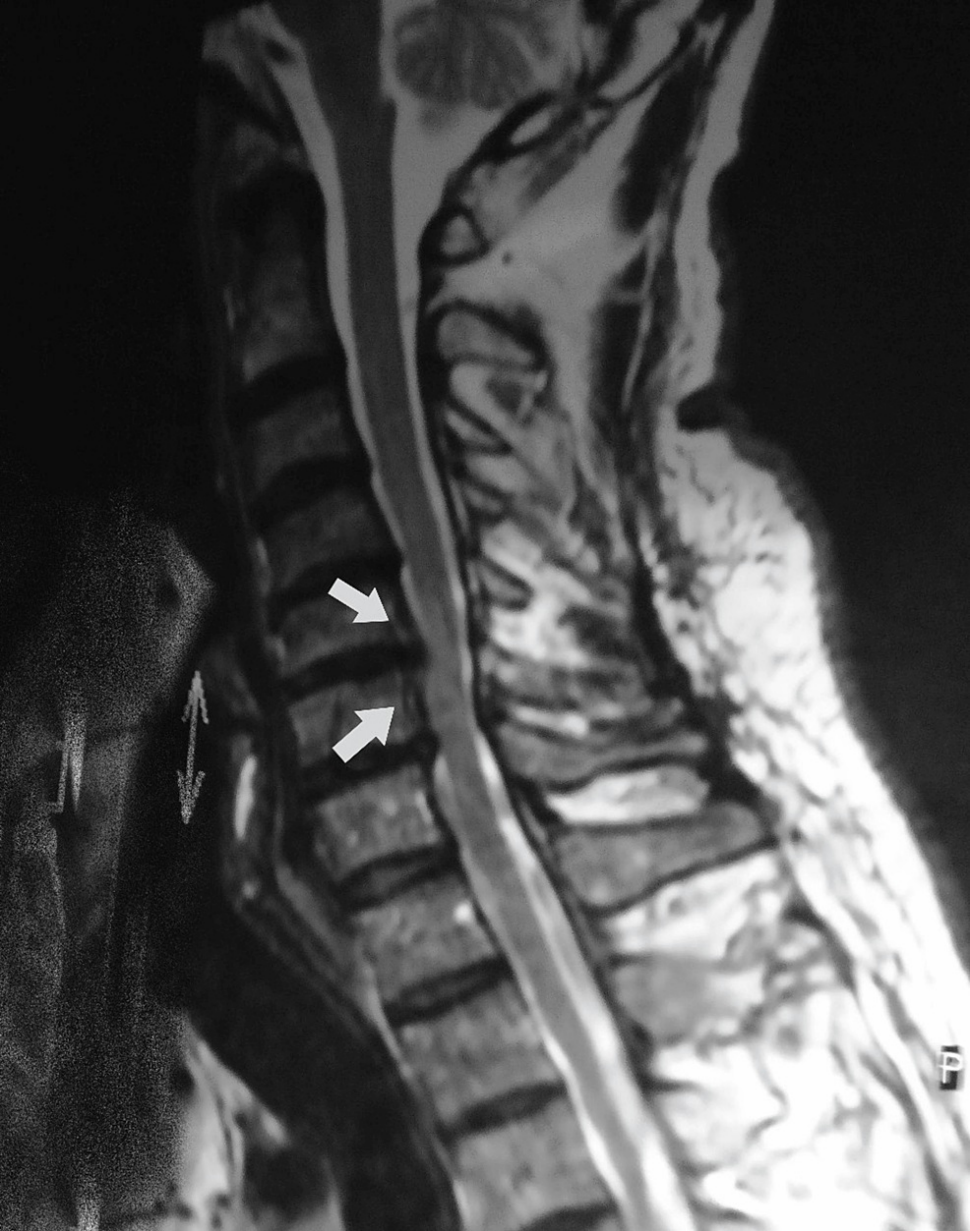
Imaging Example for Cervical Stenosis as a Potential Cause of Cervical Radiculopathy Cervical radiculopathy is caused by cervical nerve root compression and/or irritation. The arrows in the picture indicate a region of cervical stenosis, which is frequently degenerative in nature, can be found in the central canal and lateral intravertebral foramen, and is associated with cervical radiculopathy. Used by authors under the Creative Commons License 4.0.

The patient is a 49-year-old female who initially presented to PT with a chief complaint of subacute cervical pain with left upper extremity radicular symptoms for four months. Her radicular symptoms included left upper extremity pain, numbness, and weakness. The patient’s symptoms limited her from performing self-care tasks such as dressing, opening jars, grasping objects, and performing heavy household chores such as shoveling snow. The patient reported that she tried to self-manage these symptoms with stretching and yoga, but was unable to get relief. Pain medication included intermittent use of non-steroidal anti-inflammatory drugs without significant relief. Of importance, the patient demonstrated moderate fear and anxiety regarding her symptoms and the possibility of physical therapy worsening her overall condition. See Figure 1 below for an imaging example of cervical stenosis, a potential cause of cervical radiculopathy due to regional spinal degenerative changes in middle-aged women.

Review of systems and red flag screening was performed with the following results: the patient has a recent history of skin cancer, type I diabetes, lightheadedness, difficulty swallowing, and numbness in the toes of her right foot. Further questioning reviewed that the patient did not currently have symptoms of active cancer (fever, chills, sweats, unexplained weight loss) and was self-managing her diabetes, which she had for several decades. The tingling in the right foot had been chronic, and was perhaps related to the patient’s long-standing history of diabetes or poor footwear. The symptoms of lightheadedness and difficulty swallowing were later examined for relation to upper cervical instability, and significant upper cervical pathology was ruled out as noted in more detail below.

Posture observation revealed mild forward head posture and rounded shoulders bilaterally. The Alar Ligament Instability test and the Sharp-Purser test were negative. Cervical range of motion (ROM) measurements revealed good overall motion and were recorded in Table 1. Other important ROM testing revealed limited thoracic spine ROM, especially extension. Manual muscle testing was performed in sitting and results are listed in Table 1. Reflexes for the biceps, brachioradialis and triceps were performed next in sitting with a diminished response bilaterally with no significant difference noted between sides. Joint mobility via palpation was assessed in supine and prone with the following impairments noted: mild restriction in the upper and mid-cervical spine and moderate restriction noted in the thoracic spine and bilateral rib cage. To promote best practice, a cervical radiculopathy clinical prediction rule (CPR) was used [12]. The results of the clinical prediction rule can be seen in Table 2. The Neck Disability Index (NDI) was used to subjectively track the patient’s neck symptoms and impact on daily life. The patient reported a raw score of 15 which indicates moderate disability on the NDI (moderate disability = 15-24) [13].

**Table 1:** Key Physical Examination Findings Cervical range of motion (ROM) was taken via a cervical ROM magnetization wearable device. Strength testing was scored out of a maximum of 5 or via dynamometer (right/left). Sensation improvement was based on the patient report.

	Initial evaluation (Week 1)	Final follow-up (Week 5)
Tests and Measures		
Cervical ROM		
Flexion	80 degrees	70 degrees
Extension	60 degrees	70 degrees
Right side bend	40 degrees	40 degrees
Left side bend	50 degrees	50 degrees
Right rotation	70 degrees	70 degrees
Left rotation	80 degrees	70 degrees
Strength		
Elbow flexion	Right: 5 Left: 4+	Right: 5 Left: 5
Elbow extension	Right: 5 Left: 4+	Right: 5 Left: 5
Grip Strength	Right: 24 kg Left: 26 kg	Right: 34 kg Left: 34 kg
Pinch Strength	Right: 8 kg Left: 9 kg	Right: 9 kg Left: 9 kg
Sensation	Significant C6/C7 dermatome numbness present	90% improved
Median Nerve Tension	Positive bilaterally	Positive on left only
Neck Disability Index	15/50 (30%)	9/50 (18%)

**Table 2:** Cervical Radiculopathy Clinical Prediction Rule A clinical prediction rule for cervical radiculopathy used in the treatment of this patient with recorded results. Three or more positive test results indicate a high likelihood for cervical radiculopathy [12].

Examination Test		Result
Cervical rotation toward involved side less than 60 degrees		Negative
Upper Limb Tension Test (Median Nerve)		Positive
Cervical Distraction		Positive
Spurling’s		Negative

The following treatments were performed at the initial PT evaluation and treatment session based on patient presentation, nature, and irritability of symptoms, as well as best practice evidence outlined in the Neck Pain CPG. Middle thoracic manipulation (high velocity, low amplitude thrust) was used to address hypomobility in the patient’s thoracic spine and trigger various pain inhibition mechanisms. Rib traction bilaterally in prone and manual cervical traction were also used to decrease rib stiffness and relieve potential cervical nerve root compression. Manual techniques were followed up with the initiation of a home exercise program consisting of repeated McKenzie cervical retractions and thoracic extension self-mobilizations in sitting. The patient was educated on the use of McKenzie cervical retractions as a repeated motion exercise to promote the centralization of symptoms and the patient demonstrated good understanding. Further education in the first session also included objective findings of the examination, potential causes of neck and left upper extremity pain, and future PT plan of care.

**Figure 2:**
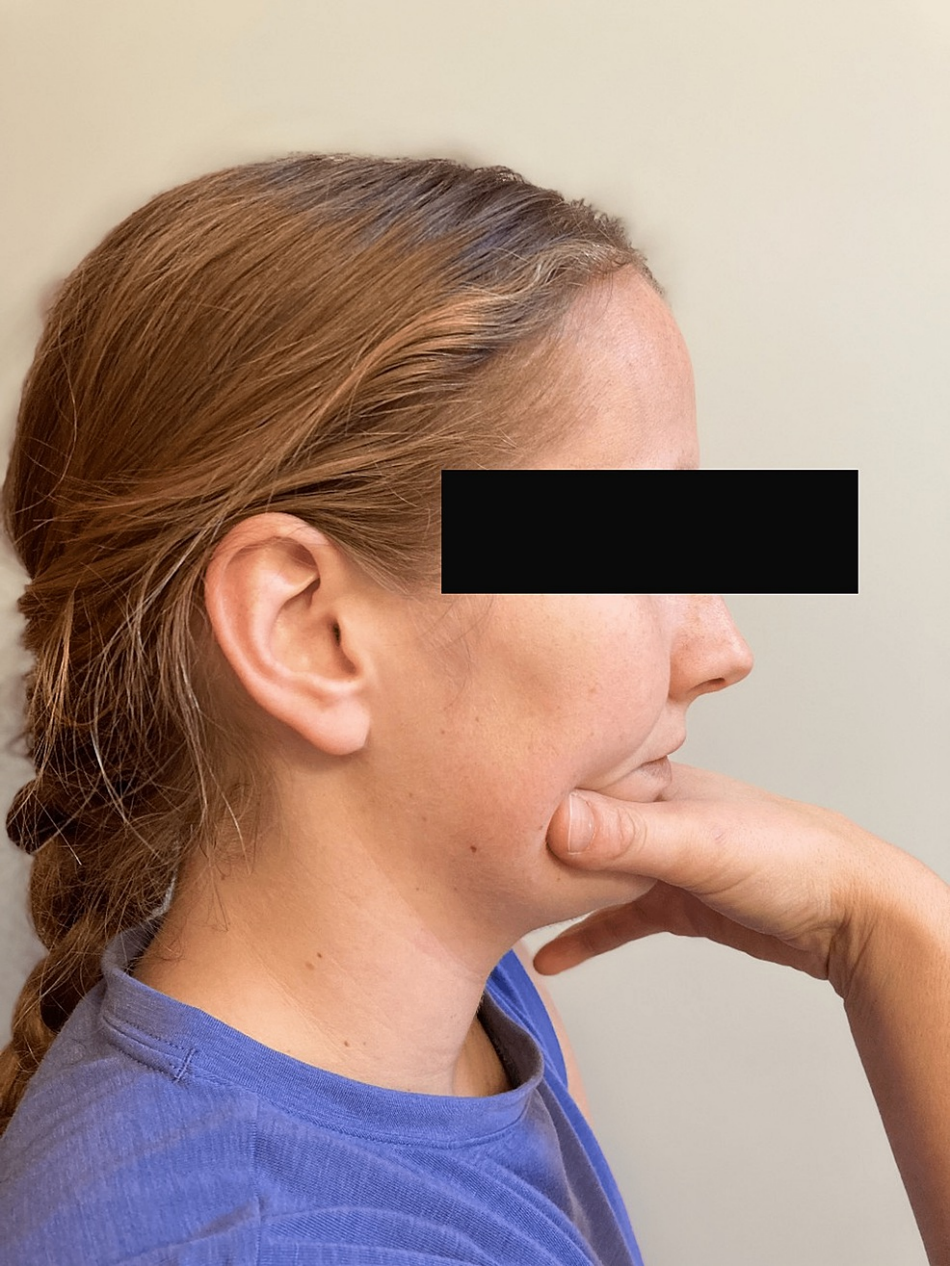
Starting Position for McKenzie Cervical Retraction Exercise with Self-Overpressure

**Figure 3:**
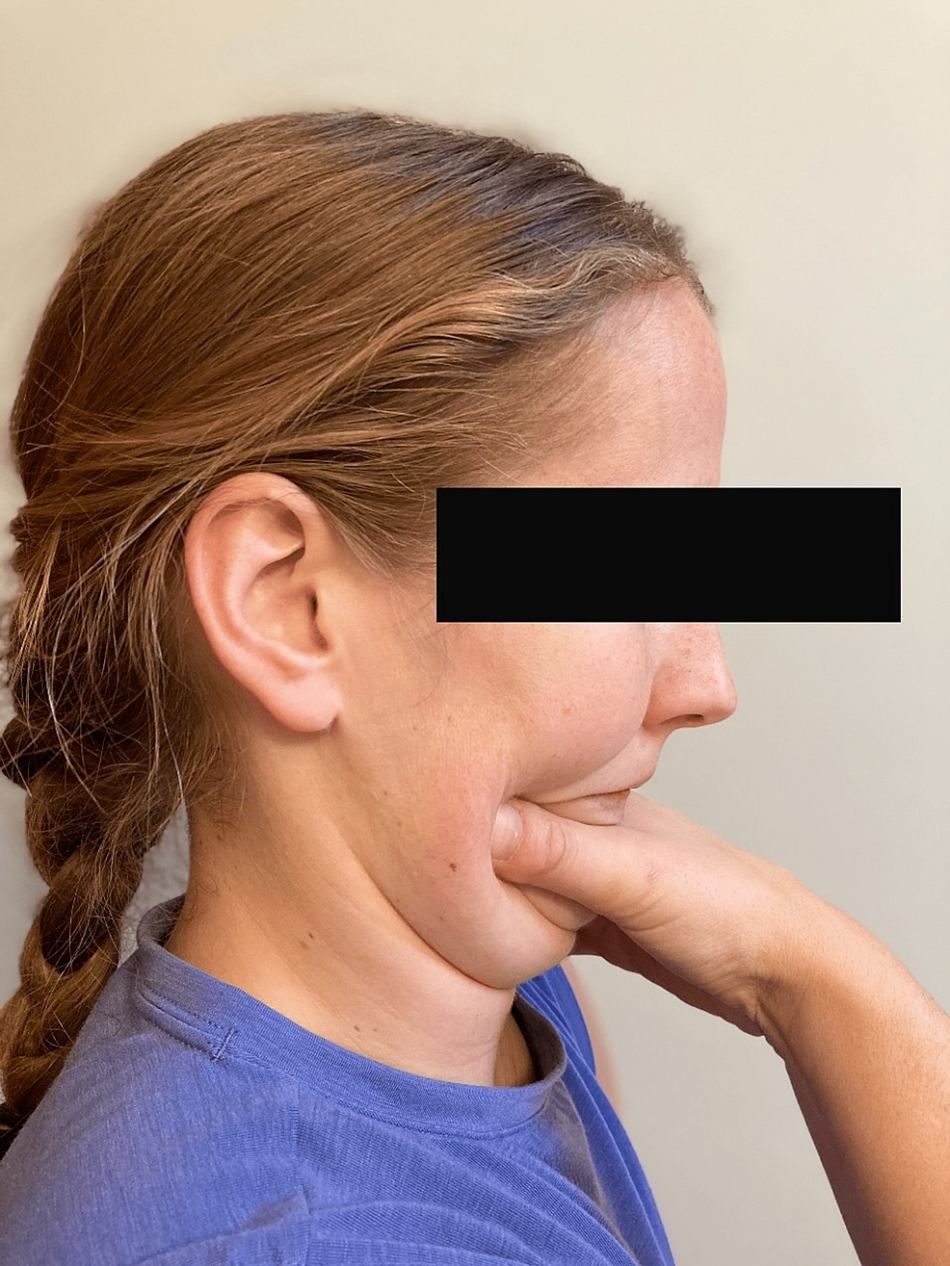
Ending Position for McKenzie Cervical Retraction Exercise with Self-Overpressure

The patient presented to the second PT treatment session on week 2 with no significant change in symptoms with neck pain and continued left upper extremity tingling/numbness down to her left index finger. Upon arrival, the patient demonstrated increased fear and anxiety behaviors regarding her symptoms. The patient had multiple concerns about her symptoms that might be red flags of more serious pathology and their relation to her current neck pain. However, upon careful questioning, red flag pathologies were ruled out, and the patient’s symptoms were likely related to a seasonal flu (fevers, chills, etc.). Manual treatment for the session included manual McKenzie cervical retractions with cervical extension to promote centralization, manual cervical traction, and manual cervical traction with median nerve glides bilaterally with an emphasis on the involved left extremity. Therapeutic exercise included progression of McKenzie cervical retraction with self-overpressure, as well as a trial of scapular retractions in sitting to promote good posture; however, scapular retractions were held due to an increase in left upper extremity pain. See Figure 2 and Figure 3 below for example of McKenzie cervical retraction with self-overpressure. Manual McKenzie cervical retractions with cervical extension resulted in centralization with the abolishment of left upper extremity symptoms except left index finger numbness. Left upper extremity symptoms returned after returning to static sitting for several minutes, but the patient was then able to again remove left upper extremity symptoms through several sets of McKenzie self-cervical retractions with overpressure. At the end of the session, the patient left the clinic with decreased neck pain and only left index finger numbness.

The patient presented to the third PT session on week 3 with a subjective rating of 5/10 for pain in her neck, left upper extremity and a new pain located in her midline thoracic spine between her shoulder blades. Scapular pain is another common manifestation of lower cervical radicular compression [14]. The patient reported that she was able to centralize her symptoms multiple times at home by use of the repeated McKenzie cervical retraction exercises with self-overpressure, but relief only seemed to last for several hours. The patient also reported that she continues to struggle with posture, and that her symptoms increased with prolonged sitting. Manual McKenzie cervical retraction techniques used on the second visit were once again utilized to promote patient centralization, but the patient experienced no significant reduction in symptoms at the end of the session. Increased attention was given at this session to educate the patient on proper posture, the role of posture in relation to symptoms, and the need to take posture breaks during prolonged sitting. The patient’s home exercise program was progressed to involve McKenzie cervical retractions with sustained self-overpressure. At the end of the session, the patient reported decreased neck stiffness, decreased pain between her shoulder blades, and only numbness in her left index finger.

At the final PT treatment session on week 5, objective measurements were taken and preparation for discharge from PT was made due to the patient's improvement. Significant week 5 examination findings and objective measures can be seen in Table 1. Upon subjective examination, the patient reported significant improvement in symptoms with only a few instances of left upper extremity symptoms over the past two weeks. The patient also stated that her constant left index finger numbness had significantly decreased and was nearly equal to the uninvolved side, and numbness only occurred with sitting and resolved with standing. No manual techniques were performed during this session due to significant need for education and postural improvement. Education included proper sitting and sleeping posture to improve symptoms, usage of McKenzie techniques for self-management as needed, positive prognosis of radicular symptoms to improve patient fear along with positive objective findings from the current session, pathophysiology of type 1 diabetes, and mechanics of nerve compression and tension during specific movements and postures. Therapeutic exercise was used to improve postural strength and endurance due to the significant relationship of symptoms and positional changes. The following exercises were performed with good technique tolerance by the patient: resisted rows in standing, bilateral shoulder external rotation in sitting, cervical retractions against a ball with scapular pinch, and standing unilateral row with a weighted pulley. The home exercise program was advanced to include bilateral shoulder external rotation and resisted rows combined with cervical retraction exercises. The patient demonstrated significant improvement in ability to manage symptoms and decreased fear regarding symptoms.

Overall, the patient presented to four PT sessions over a five-week period with McKenzie techniques, education, and/or exercises being performed at every session. Clinical outcomes were recorded throughout the episode of care and can be seen in Table 1. At the end of PT, the patient demonstrated significant improvement in left upper extremity pain, sensation, and myotomal strength, as well as a decrease in adverse neural tension in the upper extremities. The patient displayed a clinically significant decrease in her NDI score, from a raw score of 15 to a raw score of 9 (significant difference is 5 points) [13]. The patient also reported a subjective decrease in neck and left extremity pain from 5/10 to 0/10 with residual stiffness.

## Discussion

Effective examination is imperative for appropriate interventions and positive outcomes in PT. The use of CPR may lead to improvement in properly identifying cervical radiculopathy in clinical practice [15]. The specificity of CPR is 94% if three out of the four variables are present, and 99% if all four variables exist in the patient’s clinical presentation [15]. Although the patient did not fit the cluster for cervical radiculopathy, the patient did have a positive nerve tension test for the median nerve bilaterally, with the left being more irritated than the right. Potential reasons as to why the patient did not have a positive cluster for cervical radiculopathy could be related to the patient’s relatively low level of irritability and generalized hypermobility as evidenced by cervical ROM greater than 60 degrees bilaterally and the negative Spurling’s test. Individually, there is little evidence for the accuracy of special tests to diagnose cervical radiculopathy [16].

Regarding interventions, limited evidence exists concerning the most effective conservative treatments for cervical radiculopathy [8,9]. The current studies in the literature have large limitations, such as retrospective study designs and poor outcome measurements [8]. While this study represents low-level evidence, it does suggest that the addition of repeated McKenzie cervical retraction exercises can potentially lead to improvement in symptoms of cervical radiculopathy treated within a multi-modal PT program. This case report exemplifies the complete use of evidence-based practice by introducing specific repeated motion McKenzie exercises and manual techniques to a combination of conventional PT intervention for cervical radiculopathy. The three components of evidence-based practice include the use of available literature and research, clinical experience, and patient preferences. The addition of repeated McKenzie cervical motions to promote centralization was used for several reasons. First, the patient had relatively low irritability and could tolerate repeated motions without increased symptoms. This represents the clinical reasoning that is required within PT to properly match the appropriate intervention eclectically to the patient’s presentation. A current gap in the literature exists regarding whether individualized approaches are more successful than generalized approaches [8]. Secondly, the McKenzie exercises were a way for the patient to manage her own symptoms, which helped to decrease her fear and increase her confidence. The decreased reliance on therapist's direct, passive interventions promotes patient independence, self-efficacy, and empowerment for future management. Patient education was a major contributor to the success of this case report.

As best practice suggests in the 2017 revision of the Neck Pain CPG, clinicians should provide education and counseling to patients, which was especially important with this patient due to increased fear and anxiety regarding her symptoms [1]. Although this case report does not determine cause-and-effect relationship, it does suggest the benefit from repeated motion McKenzie exercises to promote centralization in patients with cervical radiculopathy. Limitations include lack of long-term follow-up, unknown contribution of additional intervention, lack of available patient imaging, as well as the contributions of the natural time course of neck pain and neck-related symptoms.

## Conclusions

Neck pain is common throughout the human lifespan, and it is also relatively common for individuals to experience signs and symptoms consistent with cervical radiculopathy. The Neck Pain CPG suggests using a multimodal approach for the most effective conservative treatment of cervical radiculopathy. Currently, there is a limited amount of evidence that determines the best practice for the evaluation and treatment of cervical radiculopathy. This case report highlights the potential for short-term improvement of patient symptoms with the addition of McKenzie repeated motion exercises to a conventional PT program. The use of sound clinical reasoning to match the nature, irritability, and severity of the patient’s problem to the best intervention likely dictates a large amount of the success of specific conservative interventions.
